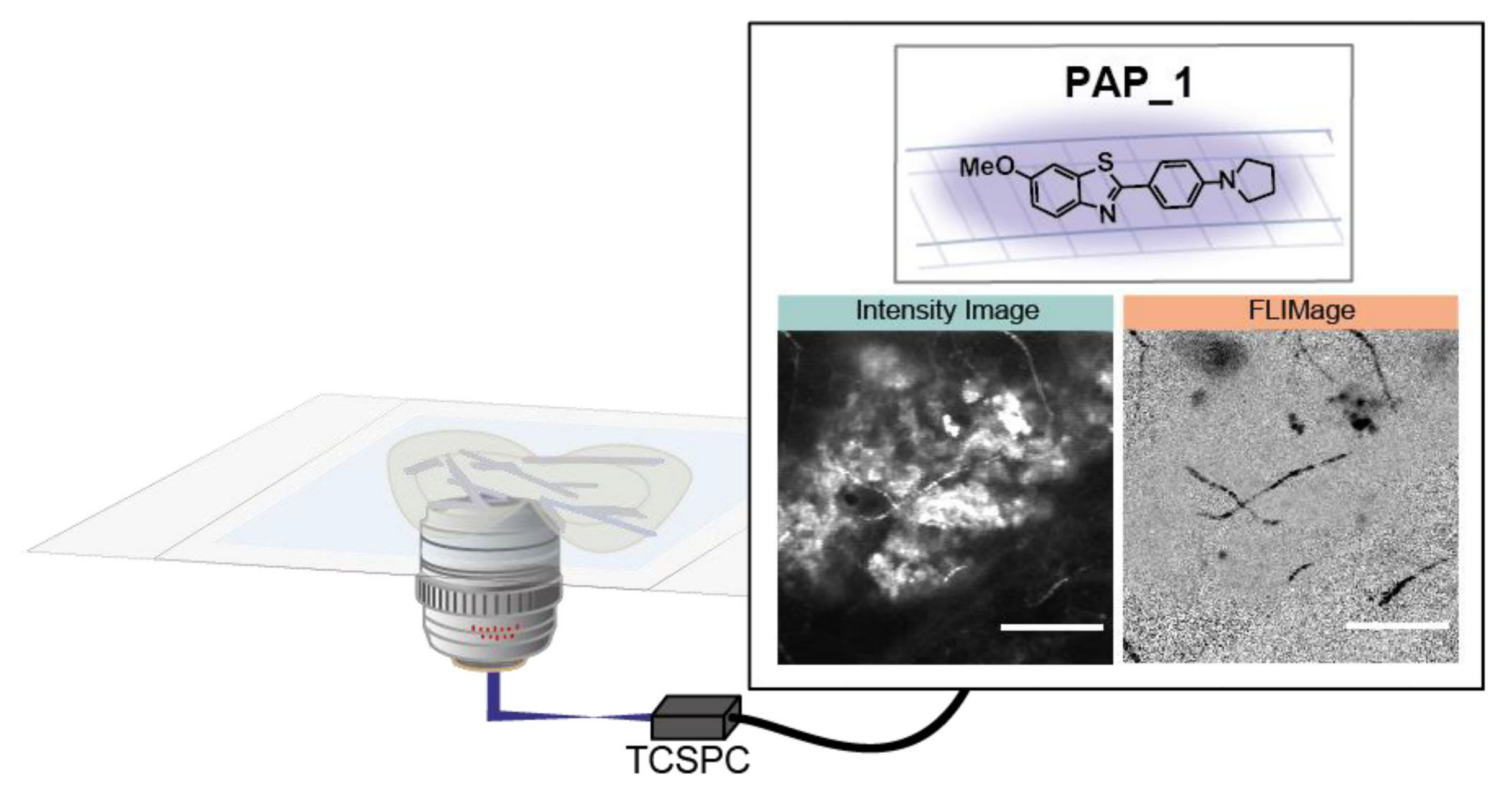# A Comparative Study of High-Contrast Fluorescence Lifetime Probes for Imaging Amyloid in Tissue

**DOI:** 10.1021/acs.jpcb.1c07762

**Published:** 2021-12-09

**Authors:** Felix Gorka, Sam Daly, Colin M Pearson, Edita Bulovaite, Yu P. Zhang, Anoushka Handa, Seth G. N. Grant, Thomas N. Snaddon, Lisa-Maria Needham, Steven F. Lee

**Affiliations:** a Yusuf Hamied Department of Chemistry, University of Cambridge, Cambridge, CB2 1EW, UK; b Department of Physics, Philipps-University Marburg, Marburg, 35032, Germany; c Department of Chemistry, Indiana University, Bloomington, Indiana, 47405, USA; d Genes to Cognition Programme, Centre for Clinical Brain Sciences, University of Edinburgh, Edinburgh, EH16 4SB, UK; e Simons Initiative for the Developing Brain, Centre for Discovery Brain Sciences, University of Edinburgh, Edinburgh, EH8 9XD, UK; f Department of Chemistry, University of Wisconsin-Madison, Madison, Wisconsin, 53706, USA

## Abstract

Optical imaging of protein aggregates in living and post-mortem tissue can often be impeded by unwanted fluorescence, prompting the need for novel methods to extract meaningful signal in complex biological environments. Historically, benzothiazolium derivatives, prominently Thioflavin T, have been the state-of-the-art fluorescent probes for amyloid aggregates, but their optical, structural, and binding properties typically limit them to *in vitro* applications. This study compares the use of novel uncharged derivative, PAP_1, with parent Thioflavin T as a fluorescence lifetime imaging probe. This is applied specifically to imaging recombinant α-synuclein aggregates doped into brain tissue. Despite the 100-fold lower brightness of PAP_1 compared to Thioflavin T, PAP_1 binds to α-synuclein aggregates with an affinity several orders of magnitude greater than Thioflavin T, thus we observe a specific decrease in the fluorescence lifetime of PAP_1 bound to α-synuclein aggregates resulting in a separation of >1.4 standard-deviations between PAP_1-stained brain tissue background and α-synuclein aggregates that is not observed with Thioflavin T. This enables contrast between high fluorescent background tissue and amyloid fibrils that is attributed to the greater affinity of PAP_1 for α-synuclein aggregates, avoiding the substantial off-target staining observed with Thioflavin T.

## Introduction

The self-association of soluble peptides into insoluble proteinaceous accumulations in the intra- and extra-cellular space of the human brain is a common physical phenomenon among neurodegenerative diseases.^1^ The detection and characterisation of these amyloidogenic protein aggregates is vital for understanding the molecular mechanisms that underlie disease pathology. Fluorescence microscopy techniques are highly suited to the study of amyloid proteins, enabling kinetic and structural characterisation of aggregates by utilising the photophysical and binding properties of the existing palette of amyloid dyes.^2^ Among these is the N-methyl benzothiazolium dye Thioflavin T (ThT), which has become a gold-standard for studying amyloid aggregation *in vitro* due to its large turn-on fluorescence response upon binding (~1000 fold).^3,4^ This phenomenon is generally explained by the molecular rotor model, resulting from competition between radiative decay of the photo-excited state and a fast non-radiative deactivation channel linked to a dihedral twist between the aromatic subunits. The latter is suppressed when bound to amyloid aggregates culminating in a large increase in fluorescence quantum yield (Φ_Fl_) and fluorescence lifetime (τ_Fl_).^5,6^ However, ThT binds amyloid with comparatively low affinity (μM) and its positive charge prevents blood-brain barrier permeability, rendering it unsuitable for most *in vivo* applications.^7^ Uncharged derivatives of ThT do not exhibit molecular rotor behaviour due to the lack of a positive charge on benzothiazole nitrogen. Instead, they behave as highly emissive fluorophores in polar solvents, but are prone to aggregation in aqueous media.^8,9^ Despite uncharged derivatives lacking the same magnitude of turn-on fluorescence upon amyloid binding exhibited by ThT; their higher affinity (nM) and ability to cross the blood-brain barrier render them useful amyloid probes.^10^

Currently, clinical amyloid imaging, for instance in diagnosis of Alzheimer’s disease, is mostly based on radiological methods like positron emission tomography (PET). Pittsburgh compound B,^11^ a neutral derivative of ThT radiolabelled with, for instance, ^11^C, has been widely used as a PET-marker for amyloid β (Aβ) and motivated further engineering of the neutral ThT scaffold to this purpose.^12^ Optical *in vivo* imaging, on the other hand, is still in its early stages of development, but shows potential to aid in understanding the underlying protein aggregation process due to its higher spatial resolution and multiple signal parameters sensitive to the microenvironment of the dye.^3^ However, fluorescence imaging of *in vivo* and *post mortem* brain tissue can be impeded by spatially heterogeneous background autofluorescence caused by a variety of reasons including emission of unbound or off-target bound dye and endogenous fluorophores,^13^ which is exacerbated in aged subjects.^14^ A variety of approaches have been reported in the literature to address this issue including; histochemical quenching,^15–18^ photobleaching,^19^ background subtraction,^20,21^ near-infrared dyes^7,22–25^ and fluorescence lifetime imaging microscopy (FLIM).^13^ FLIM is highly suited to the study of amyloid and can be used to exploit the sensitive response of the excited-state dynamics of amyloid probes to changes in their local environment. Post-processing techniques based on τ_Fl_ data and amyloid probes with a τ_Fl_ significantly different from the autofluorescence background have been applied to extract the desired signal.^13^ The near-infrared Aβ-probe CRANAD-3 has been used for *in vivo* imaging and separated from shorter-lived autofluorescence by its ~1.6 ns τ_Fl_ in a fit-free unmixing-approach.^24^ Furthermore, a pyridophenazine ruthenium(II) complex exhibited a 185 ns photoluminescence lifetime upon binding to Aβ and was able to be distinguished from short-τ_Fl_ artificially introduced background by use of a time-gating method.^26^

While a variety of extrinsic probes with potential for *in vivo* detection of aggregates have been developed,^27–30^ new strategies that prioritise the detection of small, heterogeneous oligomeric species are of particular interest to the amyloid community,^31^ one approach to generate better contrast is to mitigate unwanted the background of complex sample environments. In this work we compare the performance of ThT and a recently reported novel derivative PAP_1^9^ as τ_Fl_ probes of amyloid. By specifically using the τ_Fl_ information, *i.e*. the arrival time of single photons rather than the total number of photons, we were able to increase contrast in images of recombinant fibrils of α-synuclein (αSyn), the protein associated with Parkinson’s disease, artificially doped into autofluorescent brain tissue environments. In this imaging mode the contrast was achieved via a reduction in mean τ_Fl_ of αSyn-bound PAP_1 compared to tissue autofluorescence and non-specifically tissue bound PAP_1. Furthermore, we were able to capitalize on the higher affinity of PAP_1 for αSyn aggregates compared to ThT, as well as an apparent homogenizing effect on the spatial distribution of the background in the τ_Fl_ domain. These properties combined enabled the detection of αSyn fibrils by a larger contrast in τ_Fl_ than would be possible with the conventional ThT probe.

## Materials and Methods

### Preparation of mouse brain samples

Tissue samples were prepared from either WT (HFB) or heterozygous PSD95-mEos2 (vHFB) mice expressing the mEos2 protein fused to the endogenous PSD95 protein according to a published procedure.^32^

### Preparation of αSyn aggregates

Monomeric wild-type αSyn was purified from Escherichia coli according to a literature protocol^33^ and diluted into filtered (0.2 μm syringe filter, Whatman, 6780-1302) PBS buffer with 0.01% NaN_3_ to a concentration of 70 μM. Mature αSyn aggregates were obtained by incubating the monomer solution for >96 hours in the dark at 37 °C under constant shaking at 200 rpm.

Sonicated aggregates of αSyn were produced with pulse sonication using a tip sonicator (QSonica, Q125, tip size 1/8 inch). Each pulse consisted of a 5 second on state and a 15 second off state over 3 cycles at 40% power.

### Preparation of fluorophores

Stock solutions of PAP_1 and ThT (AnaSpec, AS-88306) were prepared by completely dissolving the solid into dimethyl sulfoxide (DMSO, Sigma Aldrich, 276855) to a concentration of 10 mM. To ensure PAP_1 was completely solubilized in DMSO, it was sonicated for 10 minutes. These were stored in the dark at -80 °C. The stock solutions were diluted into filtered (0.02 μm syringe filter, Whatman, 6809-1102) PBS (pH 7.4) to the required concentration for experiments. The diluted dye solutions were then sonicated and filtered (0.02 μm syringe filter, Whatman, 6809-1102). The diluted solutions were stored in the dark at 4 °C for a maximum of a week after preparation.^2^ Reasonable academic requests for PAP_1 should be sent directly to sl591@cam.ac.uk, where upon will be happy to share.

Solutions of anti-aSyn antibody (211) labelled with Alexa Fluor 647 (Syn211-AF647) (sc12767, Santa Cruz Biotechnology) were prepared in filtered (0.02 μm syringe filter, Whatman, 6809-1102) PBS (pH 7.4) to a concentration of 70 μM and were stored in the dark at –20 °C. The stock solutions were diluted into filtered PBS to 100 nM for experiments.

### Slide preparation for FLIM

Glass coverslips were cleaned with an argon plasma (ODC-002, Harrick Plasma) for one hour. On the coverslip, a ~1 cm^2^ area was encircled with a hydrophobic barrier pen, incubated with poly-L-lysine (PLL, 70 μL, 0.1% w/v, Sigma Aldrich, P820) for 30 minutes, the excess liquid removed, and the surface washed with filtered PBS. An aliquot of the αSyn aggregation solution was diluted to an equivalent of 5 μM monomer concentration, of which 50 μL were deposited on the PLL surface, incubated for 3 minutes, and then aspirated to ensure good adherence to the PLL coated surface. The excess liquid was removed, the surface washed with PBS again and 20 μL of either PAP_1 (50 μM), ThT (5 μM) in PBS added. ThT experiments were performed at a 10-fold reduced concentration compared to PAP_1 to account for both the greater brightness and lower αSyn binding affinity of ThT relative to PAP_1. For the measurements with both HFB and vHFB brain tissue, sectioned mouse brain tissue slices on glass coverslips were thawed to room temperature and gently washed with PBS, and a ~1 cm^2^ area surrounding the tissue was encircled with a hydrophobic barrier pen. PLL was incubated on the brain tissue for 10 minutes, the excess liquid removed and washed gently with PBS. αSyn aggregates (5 μM) or sonicated αSyn aggregates (50 nM) were deposited onto the PLL, incubated for 3 minutes, then gently aspirated. The excess liquid was removed, the surface washed with PBS again and 20 μL of either PAP_1 (50 μM), ThT (5 μM), or Syn211-AF647 (100 nM) in PBS were added. An argon cleaned glass coverslip was placed on top and imaged immediately (Figure S4).

### FLIM data acquisition

FLIM was performed on a MicroTime 200 confocal microscope (PicoQuant) using time correlated single photon counting (TCSPC). A 407 nm ps-pulsed diode laser at 40 MHz repetition rate was used as excitation source. The excitation beam was focused through a microscope objective lens (60 ×, UPlanSApo, NA 1.2, water-immersion, Olympus) onto the sample. The laser power was attenuated by a neutral density filter to a pulse energy of 250 fJ and average power of 1 μW at the objective to avoid sample degradation. Single point measurements without tissue were performed at tenfold radiant power. Emission light was collected through the same objective, separated with a dichroic mirror (ZT405rdc, Chroma) and passed through a 30 μm pinhole and either a 425 nm (PAP_1), 488 nm (ThT) or 640 nm (AF647) dielectric long pass filter (Chroma) onto the single-photon avalanche diode detector. Photon arrival times were recorded and histogrammed with 16 ps bin width. The instrument response function was measured experimentally from the KI-quenched fluorescence decay of fluorescein in basic ethanol to be of approximately gaussian shape with ~130 ps FWHM.

### Analysis of τ_Fl_ decays

Least squares fitting of the τ_Fl_ images was performed in SymPhoTime software. At first, the sum of all pixels was fitted with a double exponential tail-fit starting ~0.8 ns after the leading edge of the laser pulse to get the two major τ_Fl_ contributions. After that, a pixel-by-pixel fit was performed with fixed τ_Fl_ to recover their respective amplitudes in each pixel. The average τ_Fl_ was obtained by intensity weighting of the respective τ_Fl_ components τ_1_ and τ_2_ with amplitudes α_1_ and α_2_.^34^

$$\tau_{Fl}=\frac{\alpha_{1}\tau_{1}^{2}+\alpha_{2}\tau_{2}^{2}}{\alpha_{1}\tau_{1}+\alpha_{2}\tau_{2}}$$

Pixels with less than 50 counts were omitted for subsequent statistical analyses. Fluorescence lifetime histograms were generated from the image pixels with 0.0625 ns bin width.

## Results and Discussion

**Figure 1:**
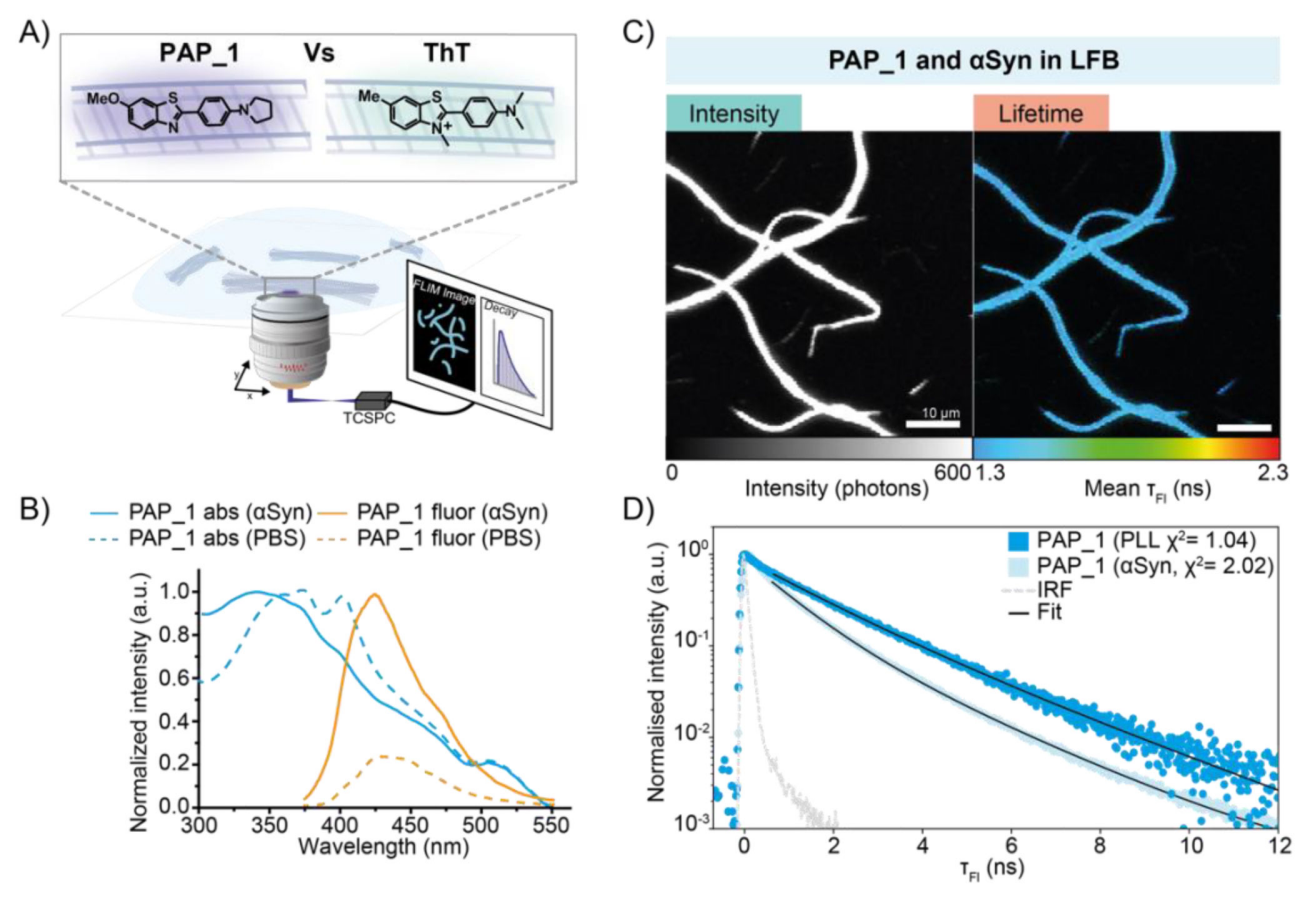
**A)** Molecular structures of PAP_1 and ThT and a schematic showing PAP_1-stained recombinant aggregates of αSyn immobilised on a low fluorescence background (LFB) PLL surface. Excitation is achieved in a confocal geometry with fluorescence photons being collected by the same objective, focussed through a pinhole and onto a single-photon sensitive photodiode. **B)** Normalized absorption and fluorescence emission spectra of PAP_1 in PBS buffer and bound to late-stage aggregates of α-Syn, reprinted from ^9^ with permission. **C)** Intensity and color-coded τ_Fl_ FLIM measurements of PAP_1 stained αSyn fibrils. **D)** Comparison of fluorescence decay curves of PLL-control and αSyn bound PAP_1 from single-point measurements with elevated integration time. τ_Fl_ values and errors were determined from a mean and standard-deviation of ≥ four single-point measurements with the same bi-exponential tail-fitting model used for image analysis.

We introduce neutral derivative of ThT, PAP_1 (Figure 1A, Figure S1A), as a τ_Fl_ imaging probe. PAP_1 is a small, weakly-fluorescent molecule that binds to mature αSyn fibrils with several orders of magnitude higher association constant than ThT (Figure S1B) and responds optically with a modest increase in fluorescence intensity (Figure 1B).^9^ Despite the more practically complex photophysical properties of PAP_1 compared to ThT, its relatively higher binding affinity for αSyn enabled amyloid detection in both the bulk and single-aggregate regimes. Initially we characterised the τ_Fl_ contrast obtained by PAP_1 in response to αSyn fibril binding in a low fluorescence background (LFB) environment (phosphate buffered saline, PBS). FLIM images of PAP_1 bound to mature recombinant αSyn fibrils adhered to a poly-L-lysine (PLL) coated surface, were readily visualized (Figure 1C) and single-point τ_Fl_ decay curves of PAP_1 were measured (Figure 1D). The fluorescence decay of αSyn fibril-bound PAP_1 was faster than the PLL control with 0.5 ns and 1.1 ns (FWHM) respectively. Bi-exponential tail-fits yielded a relatively large change in the mean τ_Fl_ of 1.51 ± 0.07 ns (for αSyn fibril-bound PAP_1) and 1.93 ± 0.06 ns for the control (PAP_1 in the absence of fibrils), a 28% reduction in τ_Fl_. The low χ^2^-values and weighted residuals (Figure S2B) suggest that the fluorescence decay after ~0.8 ns of both free and αSyn bound PAP_1 is well described by the bi-exponential model. FLIM confirmed that the 1.51 ns mean τ_Fl_ of αSyn fibril-bound PAP_1 remains uniform on the aggregate surface (Figure 1C). The magnitude of the change in measured τ_Fl_ of αSyn bound PAP_1 relative to PAP_1 in PBS does not correspond to the change in Φ_Fl_, which highlights both the complexity associated with quantifying physical properties of heterogeneous systems and the importance of spatially resolved, high-resolution measurements. Single-point decay curves of ThT on a PLL surface showed decays faster than the instrument response function thus could not be fit, however FLIM measurements (Figure S3B) and single-point decay curves of ThT bound to αSyn fibrils exhibited an increase in mean τ_Fl_ to 2.12 ± 0.1 ns (Figure S3C). Despite undergoing an increase in fluorescence intensity upon αSyn fibril incorporation, the τ_Fl_ of PAP_1 decreased upon binding, contrary to ThT. The origin of this is not yet understood, however it is known that the mechanism of fluorescence intensity increase upon binding differs between the two dyes. Whilst the fluorogenicity of ThT evolves from restriction of conformational freedom, a decrease in the aggregation state of the PAP_1 in the more hydrophobic protein environment likely drives the fluorescence increase (Figure S1C). Our previous work has shown that fitting fluorescence decays by iterative reconvolution and a stretched exponential decay law models the change in τ_Fl_ upon binding well in single-point measurements.^9^ However, this method was found too flexible and unreliable when fitting single pixels with lower photon counts in imaging experiments. Instead, tail-fitting with a bi-exponential model is utilized and decay time amplitudes are fitted in each pixel (Materials and Methods). The decrease in PAP_1 τ_Fl_ upon binding was captured by both models.

**Figure 2:**
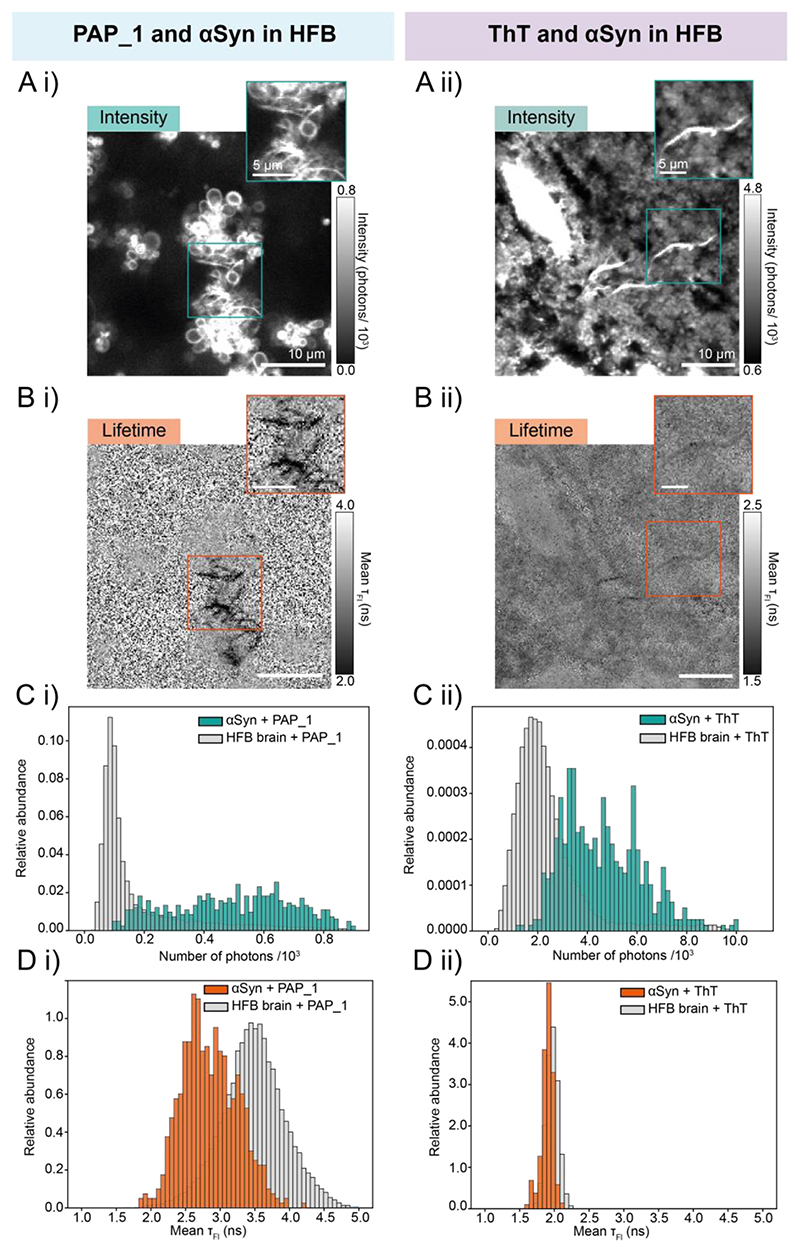
Images showing **Ai)** fluorescence intensity and **Bi)** τ_Fl_ of PAP_1 stained αSyn aggregates and **Aii)** fluorescence intensity and **Bii)** τ_Fl_ of ThT stained αSyn aggregates both imaged in a WT HFB mouse brain tissue sample. Histograms comparing the pixel values αSyn aggregates and WT HFB stained with PAP_1 in the **Ci)** fluorescence intensity and **Di)** τ_Fl_ domains and stained with ThT in the **Cii)** fluorescence intensity and **Dii)** τ_Fl_ domains. Both the intensity and τ_Fl_ contrast of PAP_1 and ThT stained images have been set independently.

We next investigated the feasibility of ThT and PAP_1 as τ_Fl_ imaging probes for αSyn aggregates in tissue. We affixed αSyn fibrils onto thin sections of adult mouse brain tissue using PLL, which were subsequently stained with either ThT or PAP_1, to simulate a high fluorescence background (HFB) (Figure S4). The fitted τ_Fl_ values obtained enable the relative contrast between HFB and αSyn to be determined. In the WT HFB samples, while some αSyn fibrils could be somewhat visually distinguished from background in the intensity domain for both dyes (Figure 2Ai-ii, Figure S5A, Figure S6A), this became increasingly difficult in regions of particularly intense tissue or off-target dye fluorescence. A mean τ_Fl_ image with PAP_1, however, provided sharper contrast between stained αSyn fibrils (<2.9 ns) and tissue background (>3.4 ns), as the convoluted intensity features of the latter were homogenized in the τ_Fl_ domain (Figure 2Bi, Figure S5B). The intrinsic ~28% decrease of PAP_1 in τ_Fl_ upon binding aided this contrast, but its 1.51 ns average τ_Fl_ was likely not obtained exactly due to mixing with tissue autofluorescence signal and poorer resolution of the decay curve at the given signal levels. The τ_Fl_ measured is a complex convolution of the probe and the tissue environment, the τ_Fl_ values should not be over-interpreted as an absolute quantification, the significance and utility arises purely from the relative contrast generated by it. Despite being able to visualize ThT stained αSyn fibrils in the intensity domain (Figure 2Aii, Figure S6A), a significantly weaker τ_Fl_ contrast was observed (Figure 2Bii), with stained αSyn fibrils showing τ_Fl_ of ~1.9 ns ns compared to background values of >1.9 ns as well as several instances of fibrils being undiscernible from background in the τ_Fl_ domain (Figure S6B).

Histograms of the image pixel values comparing both fluorescence intensity (Figure 2Ci-Cii) and τ_Fl_ (Figure 2Di-Dii) of stained αSyn aggregates and WT HFB were generated by applying a threshold to the images. Detection of αSyn aggregates in HFB tissue with both ThT and PAP_1 was severely hindered by poor contrast in the intensity domain (Figure 2Ci-Cii). While the ThT staining yielded a mean τ_Fl_ of 1.90 ± 0.09 ns at the αSyn aggregates compared to 1.97 ± 0.09 ns of WT HFB tissue and thus only minute τ_Fl_ contrast, PAP_1 staining achieved greater separation with 2.85 ± 0.40 ns on αSyn aggregates and 3.46 ± 0.45 ns of WT HFB. Thus, τ_Fl_ distributions of WT HFB background and αSyn bound PAP_1 were separated by >1.4 σ (Figure 2Ci), while ThT can only achieve a difference of 0.7 σ (Figure 2Cii). In addition, the τ_Fl_ histograms overlapped by 45% with PAP_1 and 67% with ThT staining. Consequently, PAP_1 can generate a significantly stronger τ_Fl_ contrast than parent ThT. Line profiles (Figure S7) showed spatial correlation between low-resolution intensity peaks and a >1.5 ns reduction in mean τ_Fl_ at the fibril positions. This superior contrast was aided by the more spatially homogenous background pixel values of the τ_Fl_ domain compared with the intensity domain.

The poor performance of ThT in the τ_Fl_ domain is likely due to substantial off-target binding of the dye to tissue structures, which contributed to the HFB signal with a similar τ_Fl_ as the fraction of ThT bound to αSyn. Simply put, the promiscuity of ThT for the numerous available binding sites in complex environments was larger than that of PAP_1. Despite the greater fluorescence contrast afforded by ThT in the intensity domain, the advantage was nullified by the poor specificity, preventing conclusive discrimination between amyloid aggregates and other bright structures (Figure S6A, Figure S7Aii).

In control experiments without αSyn aggregates, significantly smaller mean τ_Fl_ were observed for ThT stained (1.92 ± 0.08 ns) than unstained tissue sections (3.47 ± 0.53 ns), showing that tissue fluorescence was dominated by ThT emission in this case (Figure S8-9). Perturbation of the tissue τ_Fl_ by PAP_1 was found to be less pronounced (3.56 ± 0.64 ns). The significantly smaller dissociation constant of PAP_1 for αSyn aggregates was a clear advantage over ThT, whose off-target binding compromised the τ_Fl_ contrast.

The application of extrinsic dyes to amyloid such as PAP_1 offer labelling simplicity, environmental sensitivity, and a route toward development of blood-brain barrier penetrable probes for *in vivo* imaging; however, antibodies provide target specificity in conjunction with access to a palette of bright fluorescent probes in desirable spectral regions. The ability to detect increasingly small amyloid aggregates in increasingly complex environments is a key challenge faced by the amyloid community.^31^ Thus, we compared the capabilities of PAP_1 (Figure S10) and traditional antibody staining using the anti-αSyn antibody (syn211-AF647, Figure S11) to detect sonicated fibrils of αSyn in HFB. Bright fluorescent puncta were observed in the fluorescence intensity domain with PAP_1 stained αSyn (S10Ai). This was correlated in the τ_Fl_ domain (Figure S10Aii), and contrast was achieved in both imaging modes. However, pixel values of τ_Fl_ were significantly more homogenous compared to values of photon intensity Figure S10B). Bright fluorescent puncta were also observed when staining sonicated αSyn fibrils with syn211-AF647 in HFB (Figure S11Ai) which also manifested τ_Fl_ contrast (Figure S11Bi). However, the negative control, produced significant false positive fluorescent species, with similar contrast, when compared to directly to syn211-AF647 stained αSyn in HFB. These data suggest that antibody staining only cannot exclusively identify small αSyn aggregates (Figure S11Aii, Figure S11Ci-ii). The contrast achieved in the τ_Fl_ domain of syn211-AF647 stained αSyn is greater than the control (Figure S11Di-ii), illustrating the promise of τ_Fl_ based detection of aggregates in HFB.

Next, we endeavoured to increase the environmental complexity to explore whether the superior τ_Fl_ based detection with PAP_1 compared to ThT translated into HFB samples with spatially heterogenous emission. We created tissue sections, from genetically modified mice, which expressed a fluorescent protein conjugated to postsynaptic density protein 95 (PSD95). PSD95 is a well-studied, highly expressed protein found distributed across neurons, and found concentrated in the protein density attached to the postsynaptic membrane.^35^ This generated an additional, spatially heterogeneous source of fluorescence to simulate a complex sample matrix, that impeded detection of αSyn aggregates by fluorescence microscopy and provided a challenging test environment to compare dye properties. For consistency, this sample will be referred to as varying-High Fluorescence Background (vHFB).

Tissue autofluorescence and non-specific dye binding impeded fluorescence intensity-based detection of αSyn aggregates with both ThT and PAP_1, as with WT HFB (Figure S12Ai-ii, Figure S13A, Figure S14A). Poor τ_Fl_ contrast was observed with ThT stained αSyn fibrils in vHFB with a mean τ_Fl_ of 1.84 ± 0.16 ns at the αSyn aggregates compared to 1.92 ± 0.13 ns of vHFB tissue. PAP_1 also demonstrated a greater τ_Fl_ separation with 2.63 ± 0.44 ns on αSyn aggregates and 3.28 ± 0.27 ns of vHFB. The τ_Fl_ distributions of stained αSyn aggregates and vHFB overlap by 79% for ThT and 34% for PAP_1 (Figure S12Di-ii). As with WT HFB, line profiles (Figure S15) displayed spatial overlap between intensity peaks and a ~1 ns reduction in mean τ_Fl_ at the fibril positions in vHFB.

## Conclusion

This work has explored the performance of ThT and related, uncharged chemical probe PAP_1 in τ_Fl_ imaging of αSyn aggregates in a highly fluorescent background of brain tissue. PAP_1 exhibited excited-state kinetics which were well-approximated with bi-exponential decays and experienced a near 28% decrease in mean τ_Fl_ upon binding to αSyn aggregates compared with its unbound state. Despite PAP_1 lacking the typical molecular rotor properties of ThT and its generally low brightness, it exhibited a significantly greater contrast in the τ_Fl_ domain among the convolution of surrounding long-lived tissue fluorescence and non-specifically bound dye. Substantial off-target binding of ThT to tissue structures paired with high brightness compromised its τ_Fl_ contrast, confirming the higher binding affinity of the uncharged ThT scaffold for αSyn aggregates as a key advantage for τ_Fl_ imaging in tissue. Furthermore, τ_Fl_ contrast was aided by the more spatially homogenous features of the tissue background in the τ_Fl_ domain relative to the intensity domain. Based on these results, PAP_1 shows promise as a τ_Fl_ probe for the detection of amyloid aggregates in highly fluorescent biological environments, where intensity contrast is difficult to achieve without quenching procedures that alter the sample.

Future work involving PAP_1 for endogenous aggregate detection in the post-mortem brain is key to secure its efficacy for this application, as the obtainable τ_Fl_ contrast depends on the unwanted autofluorescence τ_Fl_. Coincident labelling with an antibody conjugated to a red fluorescent dye could, for instance, be used to confirm PAP_1’s target specificity. The promising initial results with this neutral dye provide a route towards potential alternative *in vivo* imaging with extrinsic amyloid dyes. The main limitation to the efficacy of PAP_1 as a τ_Fl_ probe is its low brightness. Therefore, in addition, further engineering of the neutral ThT scaffold towards optimized emission properties and a τ_Fl_, that also allows for contrast against shorter lived autofluorescence, will improve its performance as a τ_Fl_ probe in high autofluorescence environments such as cells and tissue, thus increasing the physiological relevance of fluorescence studies of amyloid and neurodegenerative diseases.

## Supplementary Material

Supplementary figures

## Figures and Tables

**TOC Graphic:**