# Supplementary material for: A Comparative Study of High-Contrast Fluorescence Lifetime Probes for Imaging Amyloid in Tissue
Source: J Phys Chem B. Author manuscript; Available in PMC 2024 Mar 6. (PMC7615715; doi:10.1021/acs.jpcb.1c07762)
Supplement: Supplementary figures [file EMS194355-supplement-Supplementary_figures.pdf]

Supplementary Information

A Comparative Study of High-Contrast Fluorescence Lifetime Probes for Imaging Amyloid in Tissue

Felix Gorka[a,b], Sam Daly[a], Colin M Pearson[c], Edita Bulovaite[d,e], Yu P. Zhang[a], Anoushka Handa[a], Seth G. N. Grant[d,e], Thomas N. Snaddon*[c], Lisa-Maria Needham*[a,f] Steven F. Lee*[a]

Affiliations

[a]Yusuf Hamied Department of Chemistry, University of Cambridge, Cambridge, CB2 1EW, UK
[b]Department of Physics, Philipps-University Marburg, Marburg, 35032, Germany
[c]Department of Chemistry, Indiana University, Bloomington, Indiana, 47405, USA
[d]Genes to Cognition Programme, Centre for Clinical Brain Sciences, University of Edinburgh, Edinburgh, EH16 4SB, UK
[e]Simons Initiative for the Developing Brain, Centre for Discovery Brain Sciences, University of Edinburgh, Edinburgh, EH8 9XD, UK
[f]Department of Chemistry, University of Wisconsin-Madison, Madison, Wisconsin, 53706, USA

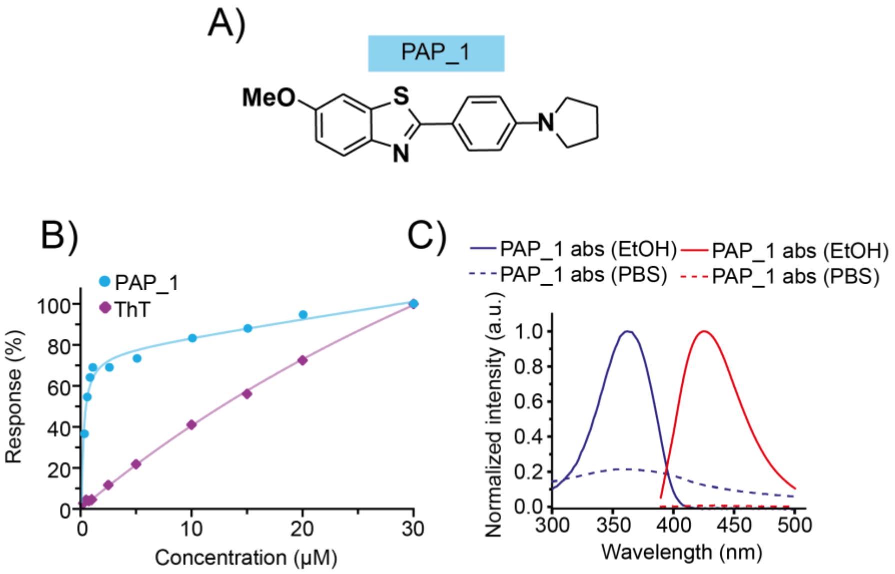

Figure S1. **A)** Structure of amyloid binding fluorophore PAP_1. **B)** Surface-plasmon resonance binding affinity curves of PAP_1 and ThT binding to late-stage aggregates of α-Syn. **B)** These data have been previously published in reference.[S1] **C)** Normalized absorption and fluorescence emission spectra of PAP_1 in PBS buffer and ethanol (EtOH).

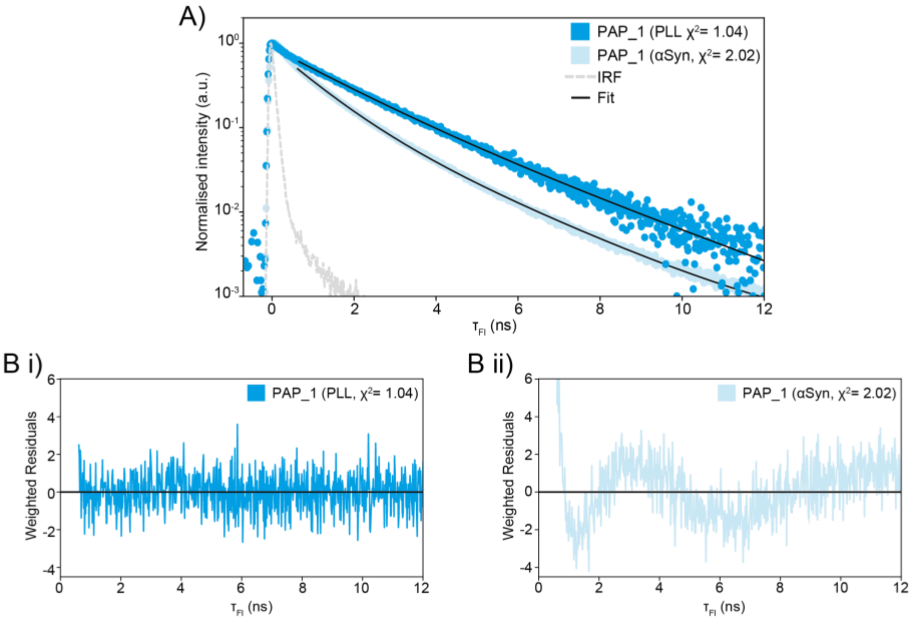

Figure S2. **A)** Recapitulation of the decay curve shown in Figure 1D and the corresponding weighted residuals versus time associated with the bi-exponential fit of **Bii)** PAP_1 on PLL and **Bii)** PAP_1 bound to αSyn fibrils.

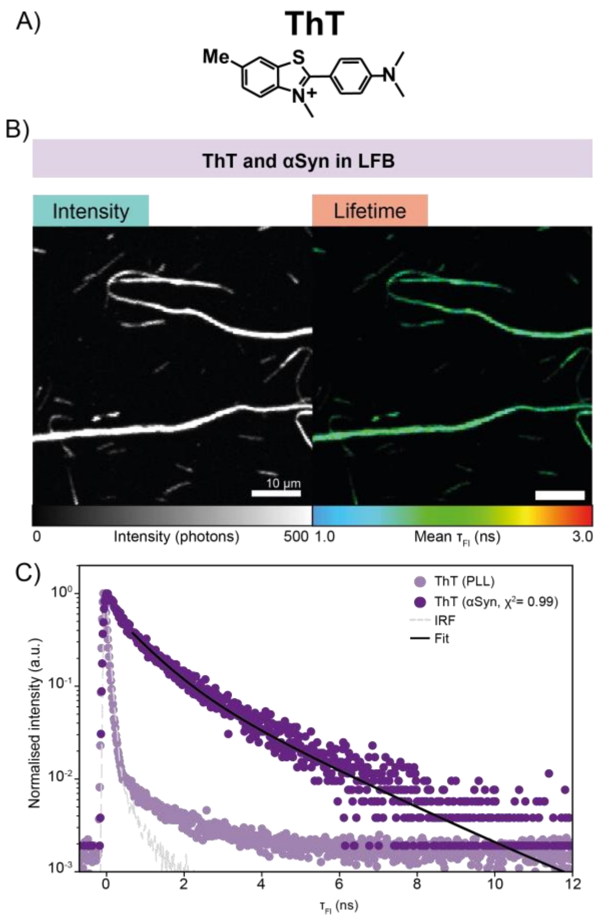

Figure S3. A) Molecular structure of ThT. **B)** Intensity and color-coded τ_{Fl} images of ThT stained αSyn fibrils. **C)** Comparison of fluorescence decay curves of PLL-control and αSyn bound ThT from single-point measurements with elevated integration time. τ_{Fl} values and errors were determined from a mean and standard-deviation of ≥ four single-point measurements with the same bi-exponential tail-fitting model used for image analysis.

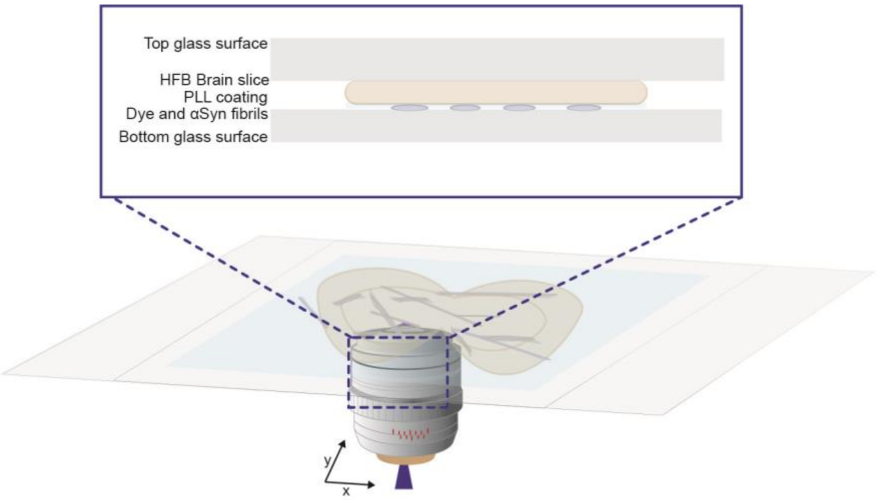

Figure S4. Schematic showing the imaging of αSyn fibrils artificially embedded in HFB brain tissue. A brain-tissue slide was coated with PLL upon which aSyn aggregates were immobilized and subsequently stained with either PAP_1, ThT or syn211-AF647. A glass coverslip was subsequently placed on top and the sample imaged. The relative thicknesses of the layers in this schematic are not to scale.

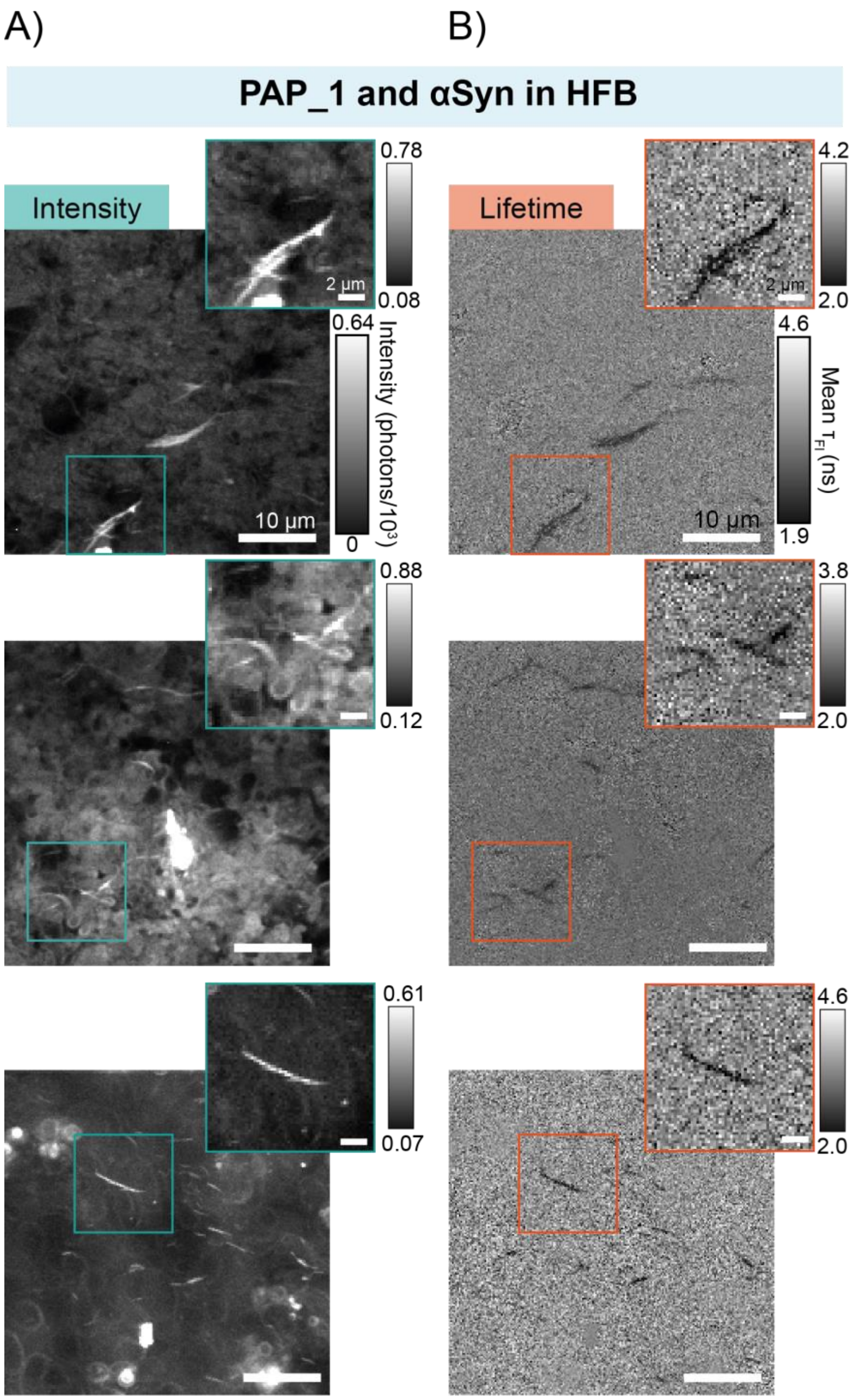

Figure S5. Images showing different regions of PAP_1 stained αSyn aggregates in HFB mouse brain tissue sample with **A)** fluorescence intensity contrast and **B)** τ_{Fl} contrast. Full view intensity and τ_{Fl} images are maintained at the same contrast (0 photons minimum, 640 photons maximum and 1.9 ns minimum, 4.6 ns maximum respectively). Magnified inset images have been set in contrast independently, values of which are shown in the respective calibration bars. Scale bars for the insets=2 µm.

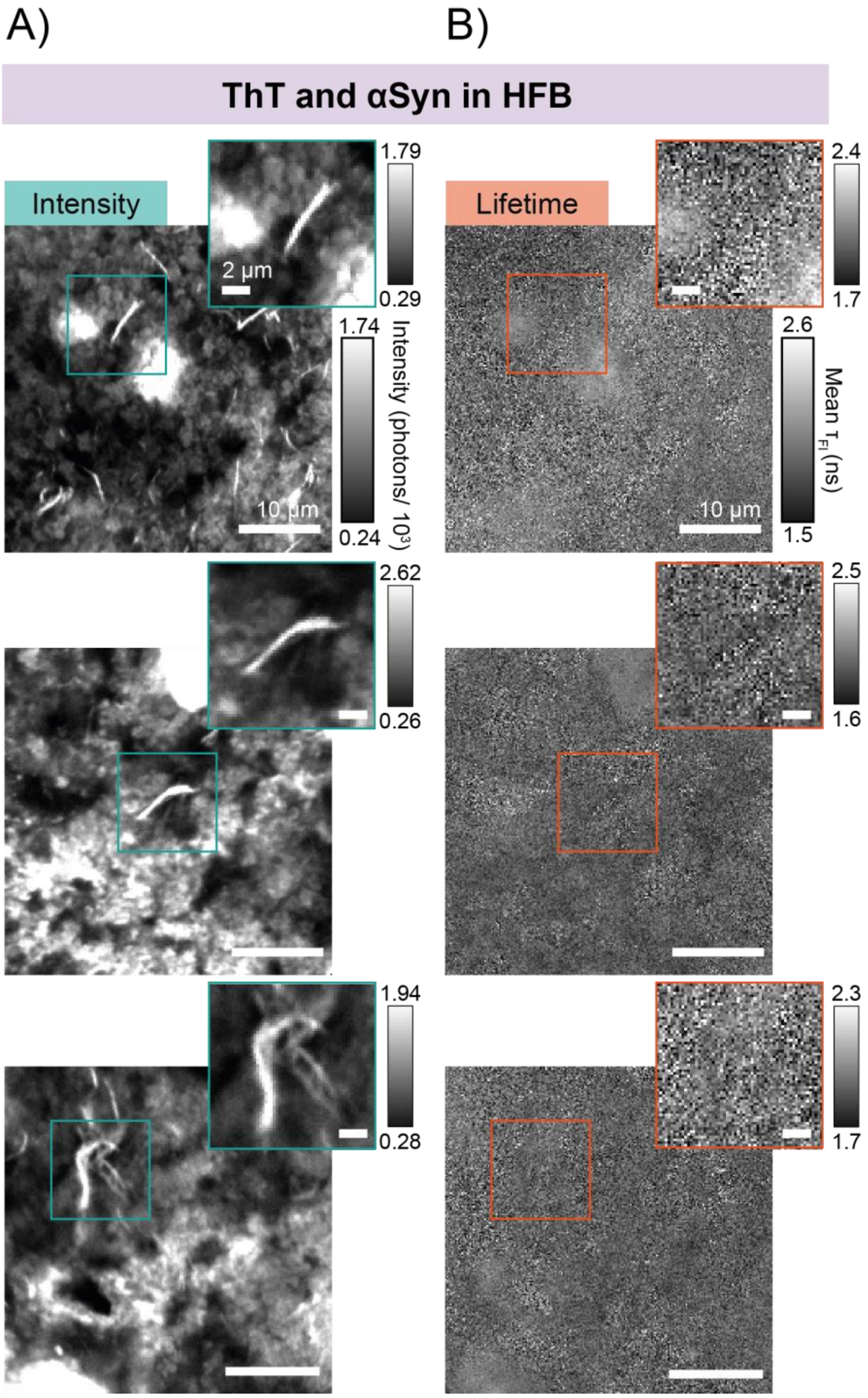

Figure S6. Images showing different regions of ThT stained αSyn aggregates in a HFB mouse brain tissue sample with **A)** fluorescence intensity contrast and **B)** τ_{Fl} contrast. Full view intensity and τ_{Fl} images are maintained at the same contrast 240 photons minimum, 1740 photons maximum and 1.5 ns minimum, 2.6 ns maximum respectively). Magnified inset images have been set in contrast independently, values of which are shown in the respective calibration bars. Scale bars for the insets =2 μm.

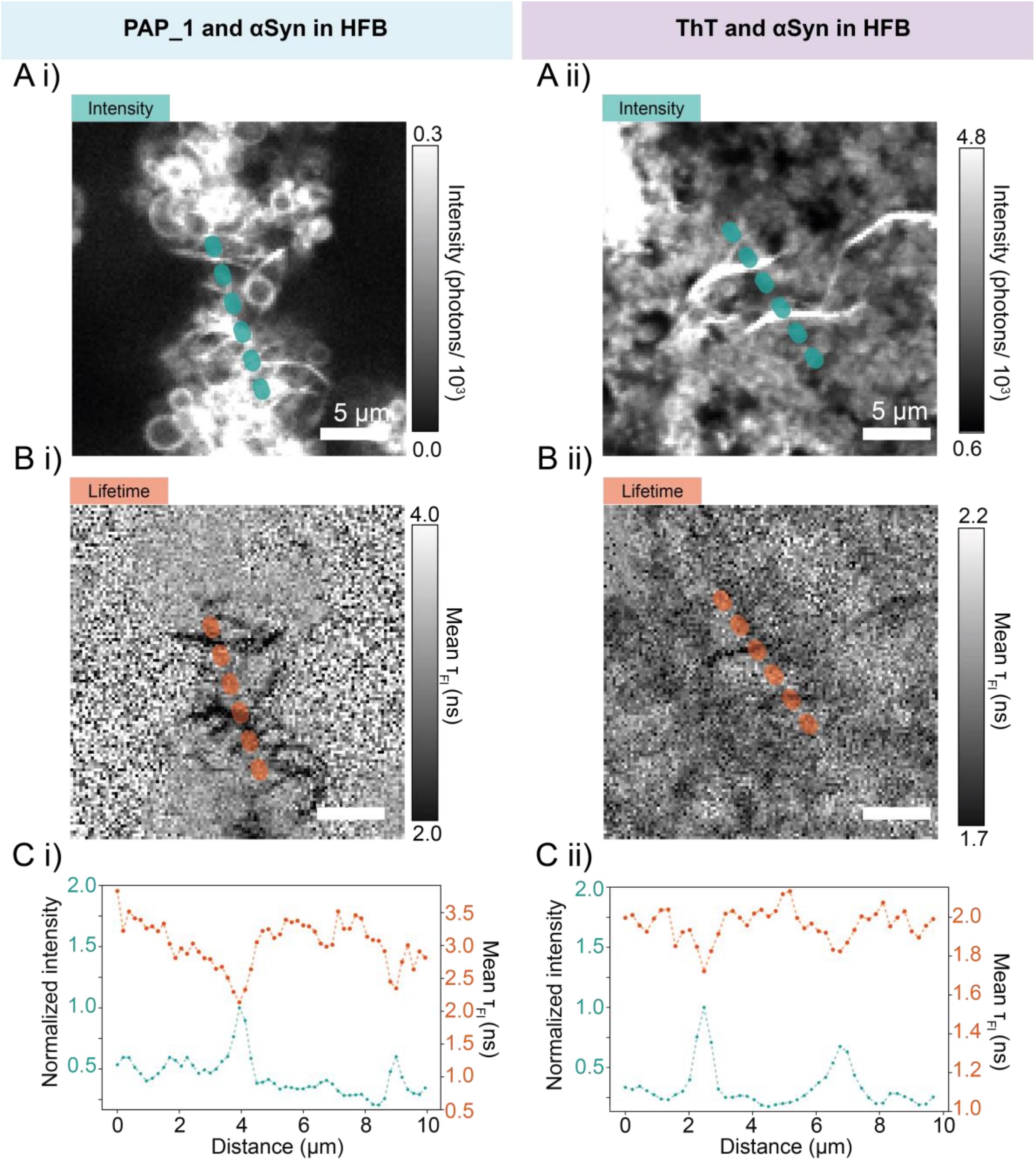

Figure S7. Component images showing **Ai)** fluorescence intensity of PAP_1 stained and **Aii)** ThT stained αSyn aggregates and **Bi)** τ_{Fl} of PAP_1 stained and **Bii)** ThT stained αSyn aggregates in HFB brain tissue. Dotted lines show spatial positions used to construct profile plots. Profile plots across **Ci)** PAP_1-stained and **Cii)** ThT-stained αSyn fibrils against the WT HFB brain tissue showing the spatial variation of intensity (blue) and τ_{Fl} (orange). Both the intensity and τ_{Fl} contrast of PAP_1 and ThT stained images have been set independently.

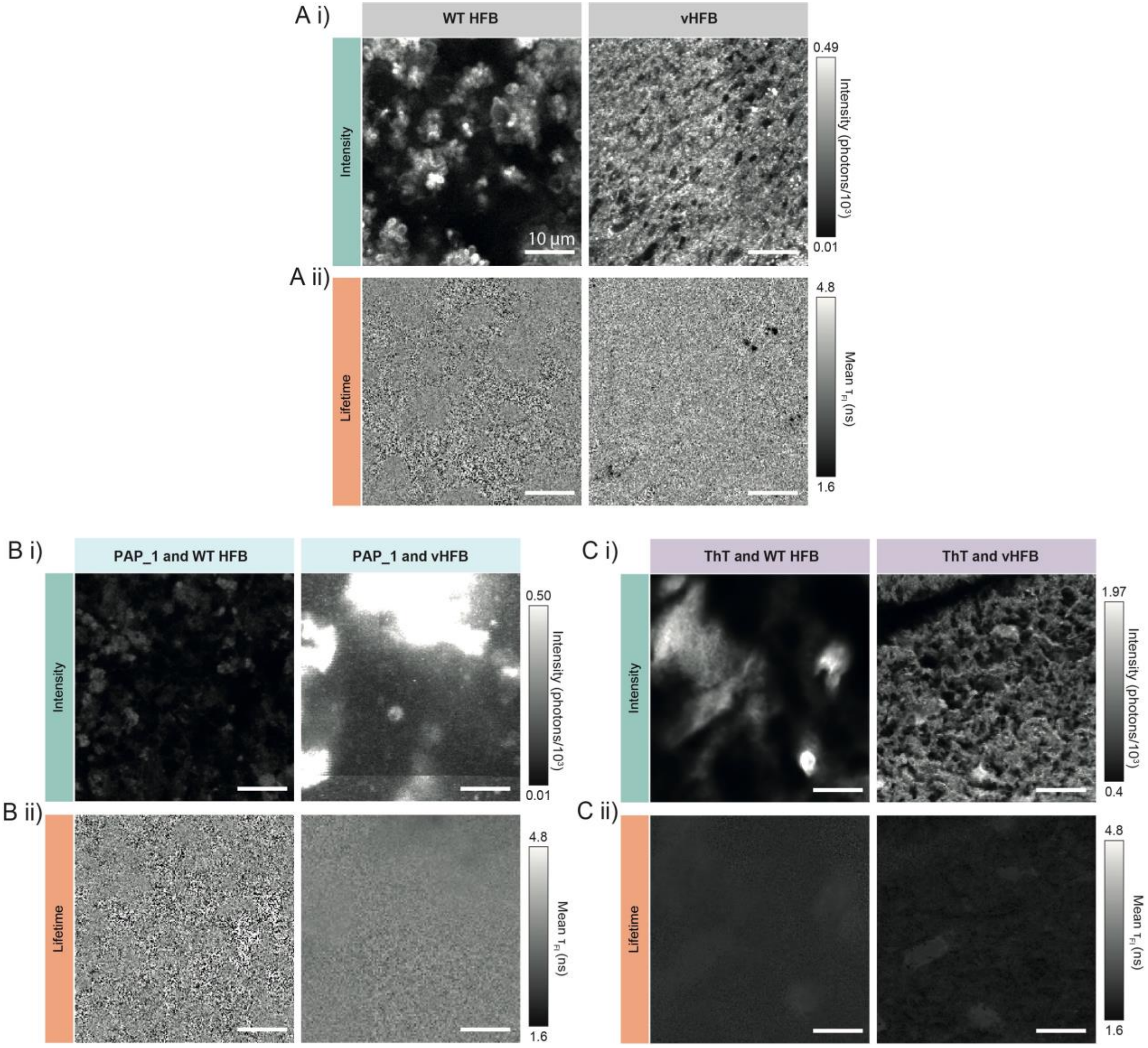

Figure S8. Control images of **Ai)** unstained WT HFB, **Aii)** unstained vHFB, **Bi)** PAP_1-stained WT HFB, **Bii)** PAP_1-stained vHFB, **Ci)** ThT stained WT HFB and **Cii)** ThT stained vHFB in both fluorescence intensity (top) and τ_{Fl} domains. Both the intensity and τ_{Fl} contrast of unstained control, PAP_1 and ThT stained images have been set independently.

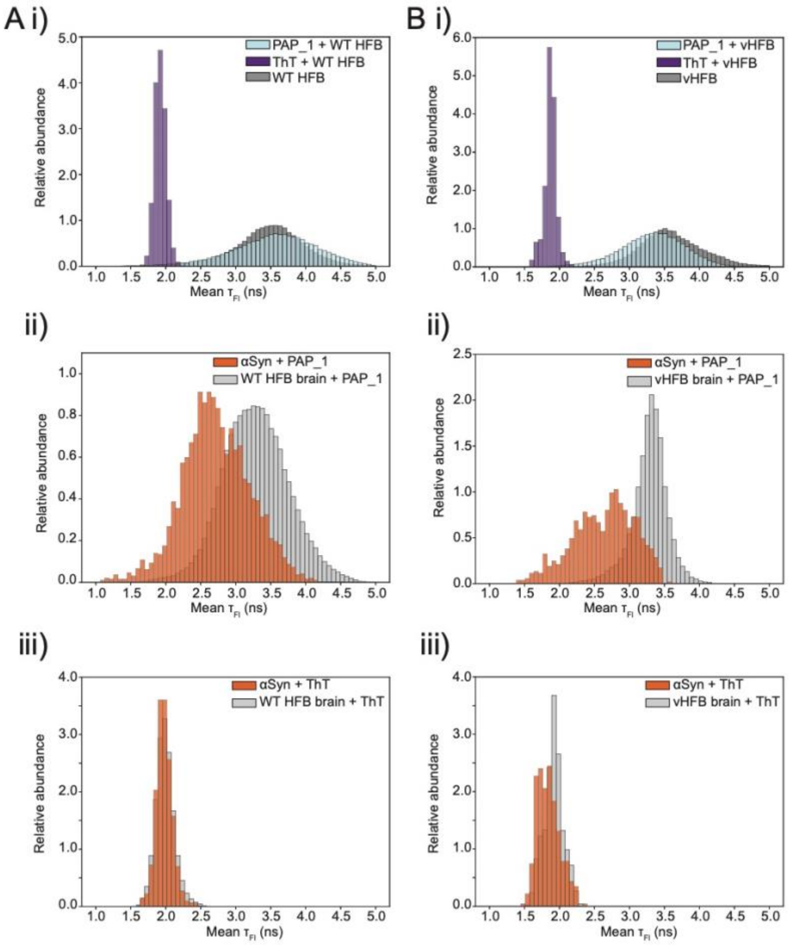

Figure S9. Histograms of τ_{FI} pixel values compiled from N ≥ 3 FLIM datasets from showing **Ai)** WT HFB controls, **Aii)** PAP_1 stained αSyn fibrils in WT HFB and PAP_1 stained WT HFB, **Aiii)** ThT stained αSyn fibrils in WT HFB and ThT stained WT HFB, **Bi)** vHFB controls, **Bii)** PAP_1 stained αSyn fibrils in vHFB and PAP_1 stained vHFB and **Biii)** ThT stained αSyn fibrils in vHFB and ThT stained vHFB.

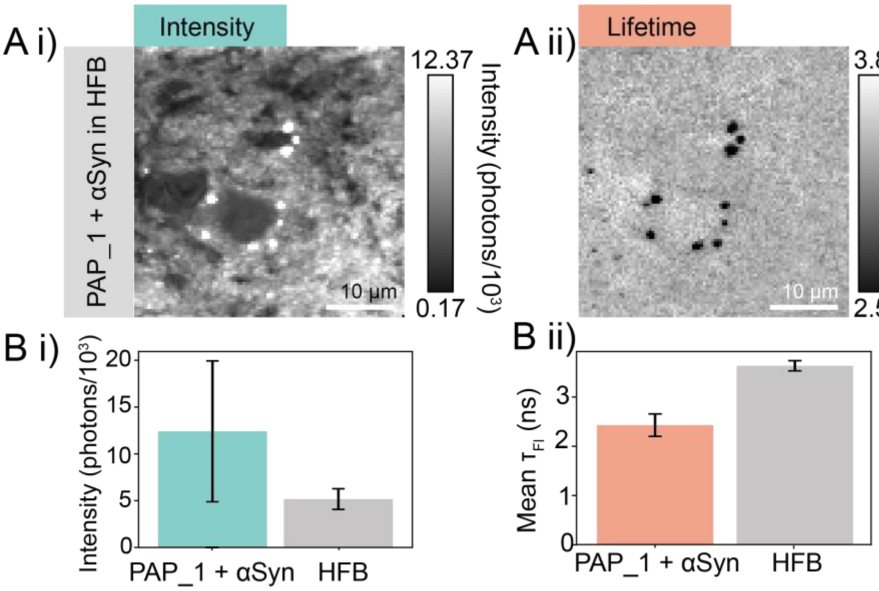

Figure S10. Images showing the of **Ai)** fluorescence intensity and **Aii)** τ_{FI} of PAP_1 stained sonicated αSyn fibrils in HFB. Bar graphs showing the mean and standard deviations of pixel values above and below a constant threshold in the **Bi**) fluorescence intensity and **Bi**) τ_{FI} domain of PAP_1 stained sonicated αSyn fibrils in HFB in order to illustrate the achieved contrast in each imaging mode upon binding to small aggregates in complex background.

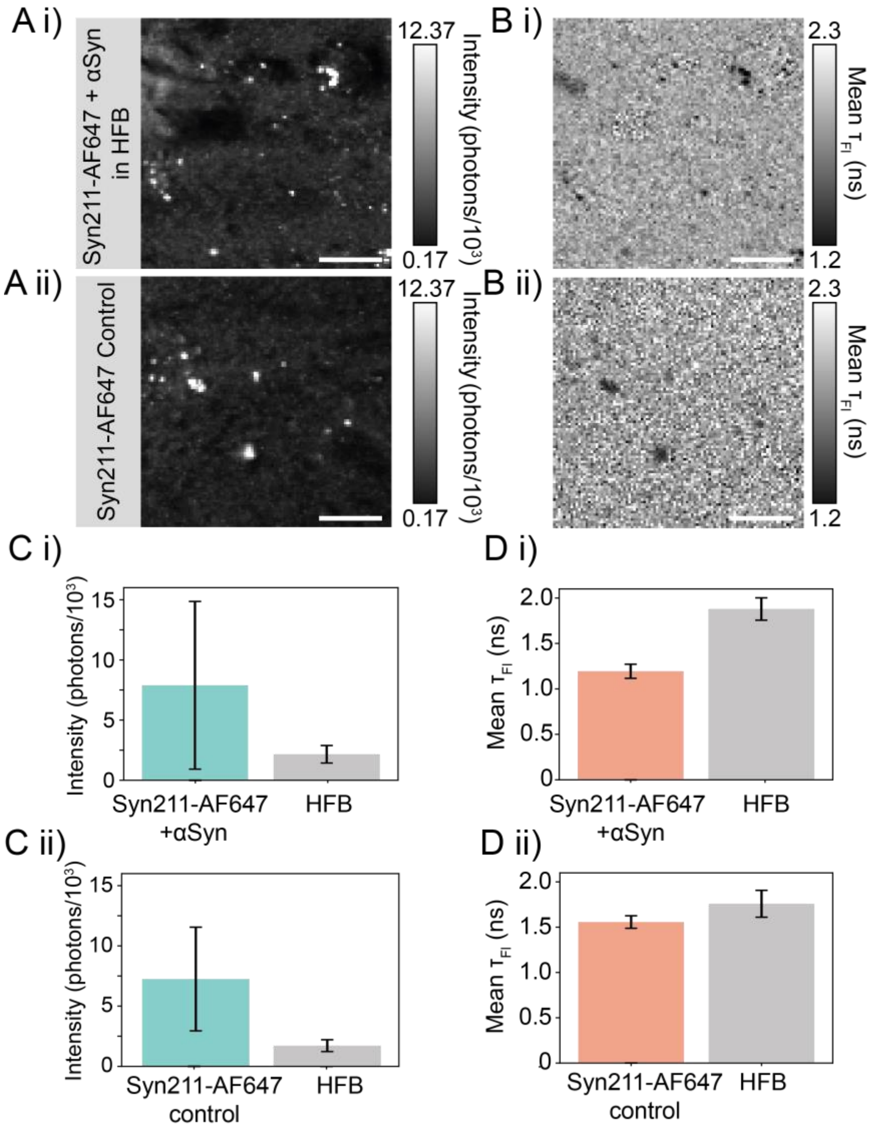

Figure S11. Images showing the **Ai)** fluorescence intensity and **Bi)** τ_{FI} of sonicated fibrils of αSyn stained with Syn211-AF647 in HFB as well as the **Aii)** fluorescence intensity and **Bii)** τ_{FI} of Syn211-AF647 in HFB as a control. Bar graphs showing the mean and standard deviations of pixel values above and below a constant threshold in the **Ci)** fluorescence intensity and **Di)** τ_{FI} domain of sonicated fibrils of αSyn stained with Syn211-AF647 in HFB and **Ci)** fluorescence intensity and **Di)** τ_{FI} domain of the Syn211-AF647 in HFB control.

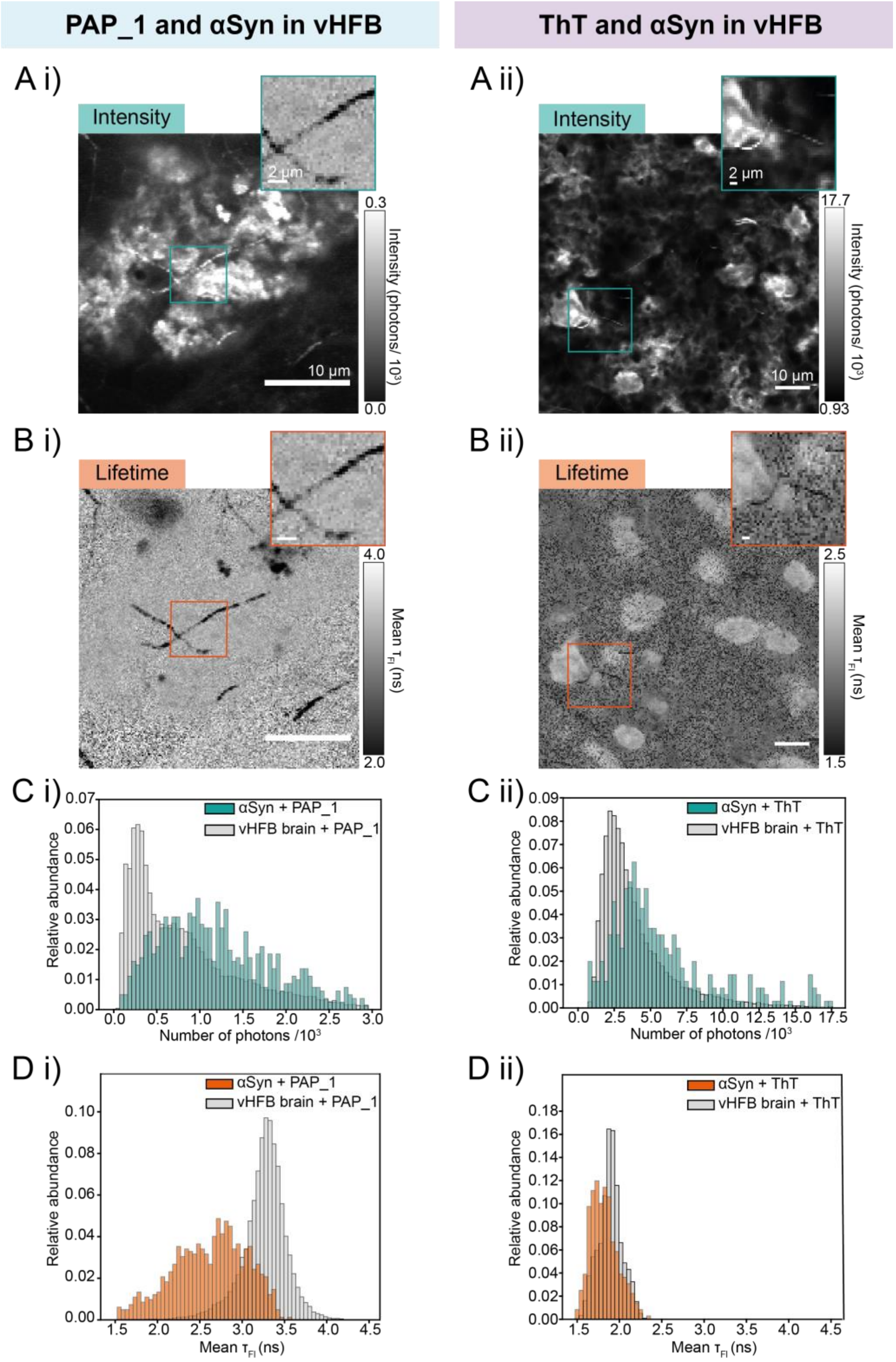

Figure S12. Images showing **Ai)** fluorescence intensity and **Bi)** τ_{FI} of PAP_1 stained αSyn aggregates and **Aii)** fluorescence intensity and **Bii)** τ_{FI} of ThT stained αSyn aggregates both imaged in vHFB mouse brain tissue sample. Histograms comparing the pixel values αSyn aggregates and vHFB stained with PAP_1 in the **Ci)** fluorescence intensity and **Di)** τ_{FI} domains and stained with ThT in the **Cii)** fluorescence intensity and **Dii)** τ_{FI} domains. Both the intensity and τ_{FI} contrast of PAP_1 and ThT stained images have been set independently.

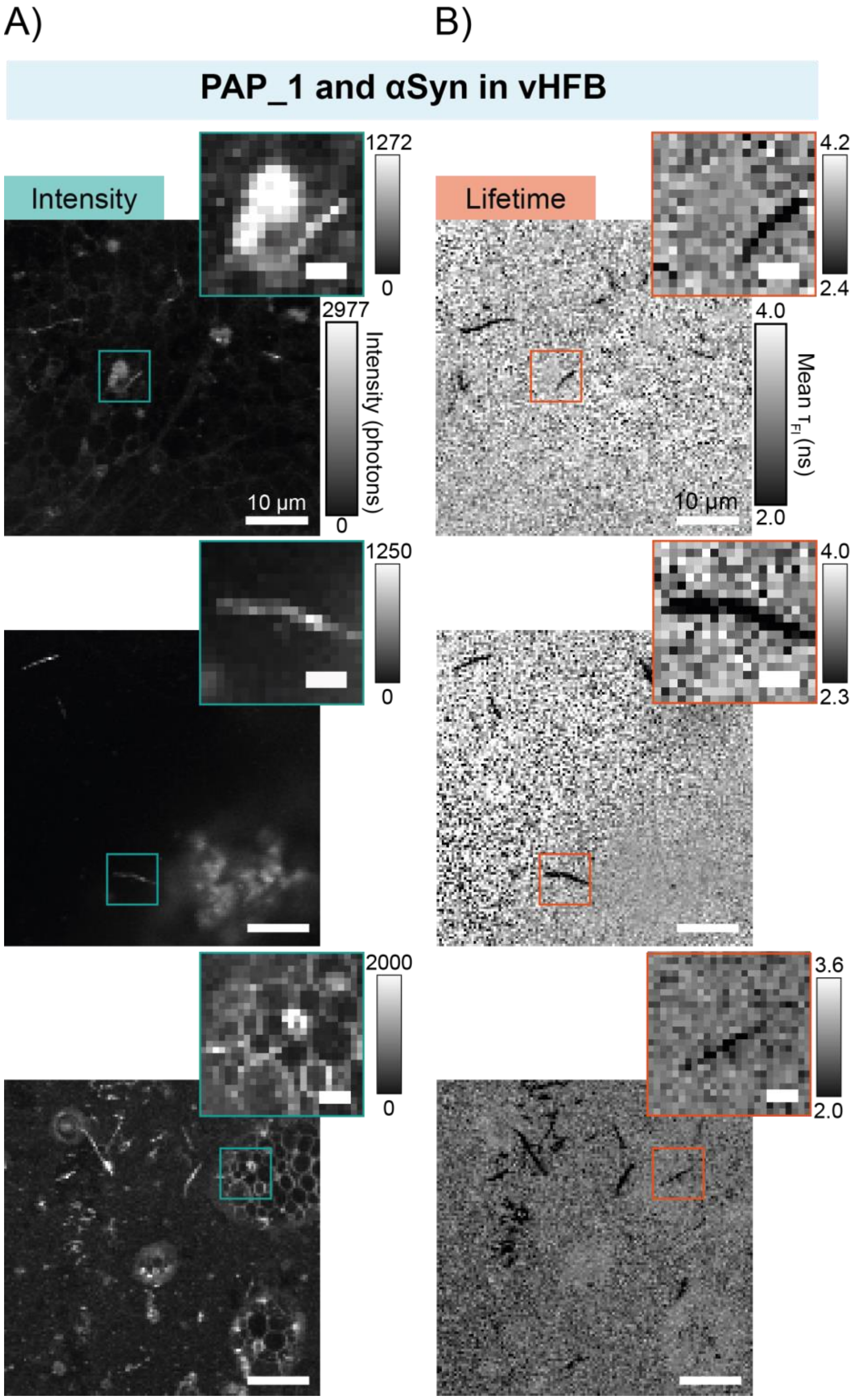

Figure S13. Images showing different regions of PAP_1 stained αSyn aggregates in vHFB mouse brain tissue sample with **A)** fluorescence intensity contrast and **B)** τ_{FI} contrast. Full view intensity and τ_{FI} images are maintained at the same contrast (0 photons minimum, 2977 maximum and 2 ns minimum, 4 ns maximum respectively). Magnified inset images have been set in contrast independently, values of which are shown in the respective calibration bars. Scale bars for the insets = 2 µm.

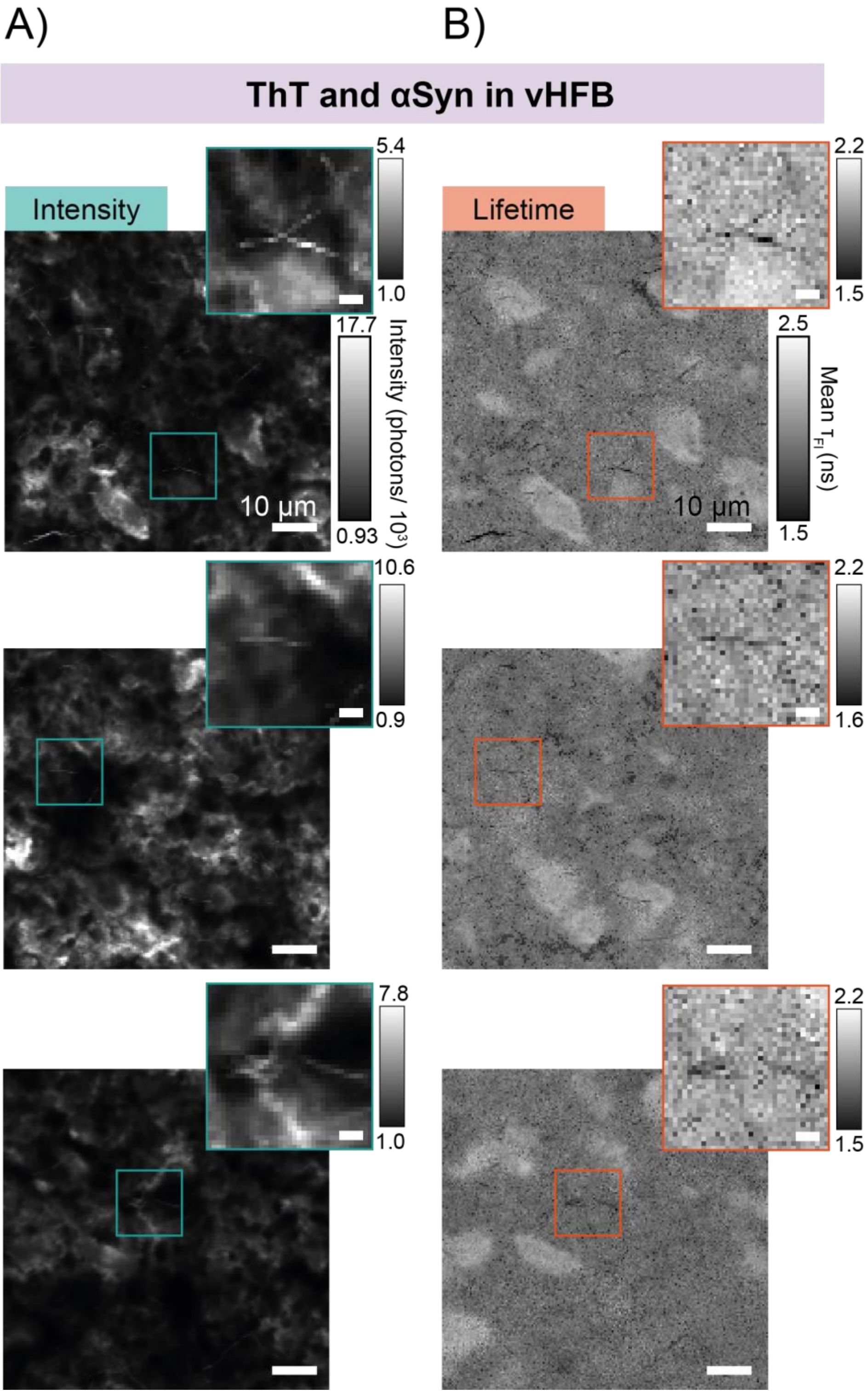

Figure S14. Images showing different regions of ThT stained αSyn aggregates in vHFB mouse brain tissue sample with **A)** fluorescence intensity contrast and **B)** τ_{Fl} contrast. Full view intensity and τ_{Fl} images are maintained at the same contrast (0 photons minimum, 2977 maximum and 2 ns minimum, 4 ns maximum respectively). Magnified inset images have been set in contrast independently, values of which are shown in the respective calibration bars. Scale bars for the insets = 2 µm.

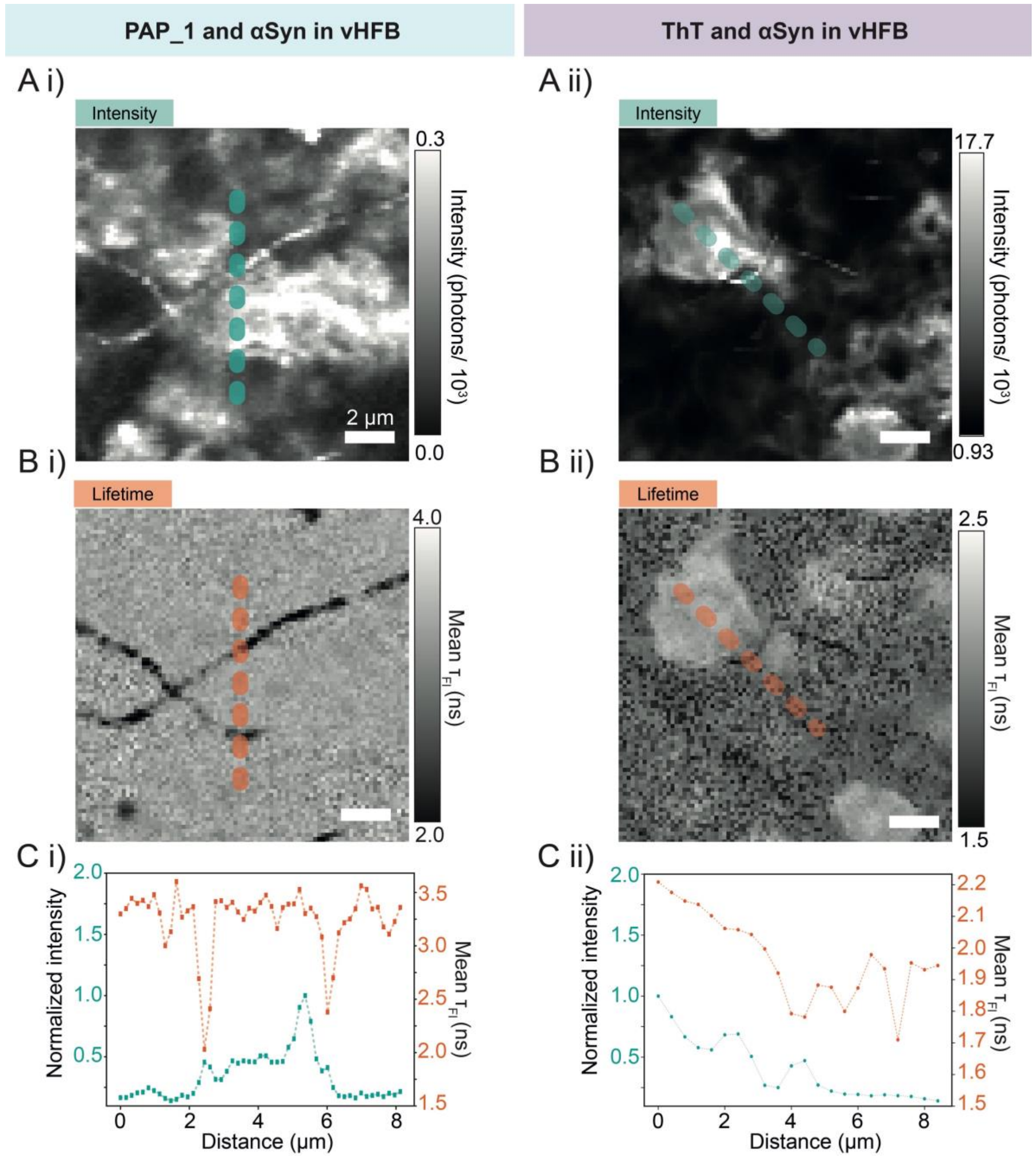

Figure S15. Component images showing **Ai)** fluorescence intensity of PAP_1 stained and **Aii)** ThT stained αSyn aggregates and **Bi)** τ_{Fl} of PAP_1 stained and **Bii)** ThT stained αSyn aggregates in vHFB brain tissue. Dotted lines show spatial positions used to construct profile plots. **C)** Profile plots across a **Ci)** PAP_1 stained and **Cii)** ThT stained αSyn fibril against the vHFB brain tissue showing the spatial variation of intensity (blue) and τ_{Fl} (orange). Both the intensity and τ_{Fl} contrast of PAP_1 and ThT stained images have been set independently.

References

(S1) Needham, L. M.; Weber, J.; Pearson, C. M.; Do, D. T.; Gorka, F.; Lyu, G.; Bohndiek, S. E.; Snaddon, T. N.; Lee, S. F. A Comparative Photophysical Study of Structural Modifications of Thioflavin T-Inspired Fluorophores. *The journal of physical chemistry letters* **2020**, *11* (19), 8406–8416. https://doi.org/10.1021/acs.jpclett.0c01549.
